# The association of burnout with work absenteeism and the frequency of thoughts in leaving their job in a cohort of healthcare workers during the COVID-19 pandemic

**DOI:** 10.3389/frhs.2023.1272285

**Published:** 2023-11-29

**Authors:** Cerina Lee, Thanh-Huyen T. Vu, John A. Fuller, Melanie Freedman, Jacqueline Bannon, John T. Wilkins, Judith T. Moskowitz, Lisa R. Hirschhorn, Amisha Wallia, Charlesnika T. Evans

**Affiliations:** ^1^ Institute for Public Health and Medicine, Center for Health Services and Outcomes Research, Northwestern University, Chicago, IL, United States; ^2^ Department of Preventive Medicine, Feinberg School of Medicine, Northwestern University, Chicago, IL, United States; ^3^ Comprehensive Cancer Center, James Cancer Hospital and Solove Research Institute, The Ohio State University, Columbus, OH, United States; ^4^ Summer Research Opportunity Program (SROP), Feinberg School of Medicine, Northwestern University, Evanston, IL, United States; ^5^ Department of Medical Social Sciences, Feinberg School of Medicine, Northwestern University, Chicago IL, United States; ^6^ Department of Medicine, Division of Cardiology, Feinberg School of Medicine, Northwestern University, Chicago, IL, United States; ^7^ Robert J Harvey Institute for Global Health, Northwestern University, Chicago, IL, United States; ^8^ Department of Medicine, Division of Endocrinology, Metabolism, and Molecular Medicine, Feinberg School of Medicine, Northwestern University, Chicago, IL, United States; ^9^ Department of Veterans Affairs, Center of Innovation for Complex Chronic Healthcare, Edward Hines, Jr. VA Hospital, Hines, IL, United States

**Keywords:** healthcare workers, absenteeism, COVID-19, burnout, psychological health

## Abstract

**Introduction:**

During the coronavirus disease 2019 pandemic, high levels of burnout were reported among healthcare workers. This study examines the association of work absenteeism and frequency of thoughts in leaving current job with burnout among a cohort of healthcare workers during the COVID-19 pandemic.

**Methods:**

A cross-sectional survey of healthcare workers was conducted from April-May, 2022 on healthcare workers from 10 hospitals, 18 immediate care centers, and 325 outpatient practices in the Chicago area and surrounding Illinois suburbs. Logistic regression models were used to assess the association of burnout scores (Oldenburg Burnout Inventory—OLBI) and its sub-scores (exhaustion and disengagement scores) with work absenteeism and thoughts of leaving work.

**Results:**

One-fifth and 60% of respondents (*n* = 1,825) reported unplanned absenteeism and thoughts of leaving their job, respectively. After adjusting for covariates, higher burnout scores, especially exhaustion scores, were associated with increased odds of unplanned absenteeism (OR = 1.04, 95% CI: 1.01–1.08). Burnout scores and both sub-scores were also positively associated with the frequency of thoughts of leaving work, e.g., each unit increase in the OLBI burnout score was associated with 1.39 (95% CI: 1.34–1.43) times higher odds of thinking about leaving work “a lot/constantly” vs. “never”.

**Discussion:**

Overall, this study cohort showed a positive association between burnout scores and unplanned work absenteeism (and frequency of thoughts in leaving job) during the COVID-19 pandemic. More research is needed to support healthcare worker well-being during times of stress and direct solutions to addressing unplanned absenteeism in the light of a pandemic.

## Background

Throughout the COVID-19 pandemic, frontline healthcare workers (HCWs) have experienced increased stress due to additional working hours, potential exposure to COVID-19, and subsequent COVID-19 related illness, all of which may have led to increased levels of burnout (1, 2). Indeed, a 2022 U.S. Surgeon General Report has highlighted the increasing rates of HCW burnout and its harmful consequences on the workforce and patient care as a result of the pandemic (3). In fact, a systematic review (4) that examined HCW and burnout during the pandemic showed that “task-related coping” was the primary strategy of coping for HCWs, in which HCWs would focus their efforts and thoughts at solving a problem to provide optimal results during stressful situations.

Previous studies (5) on HCWs have characterized burnout as having elements of emotional exhaustion (during patient contact), depersonalization (feeling detached from others), and lack of personal accomplishment (in personal skills, job success, and competency). Burnout is associated with poor quality of care and increases in medical errors, which can have dire consequences for patient safety and overall healthcare delivery effectiveness (3). Research has also found that higher levels of burnout are associated with lower levels of patient satisfaction, resulting in increased patient and family complaints (6). In addition, burnout can reduce quality of life and lead to adverse health behaviors among providers (7). Furthermore, a recent study (8) on HCWs (who had been working since before the start of the COVID-19 pandemic) reported high levels of depression and anxiety, which were significantly associated with higher levels of HCW burnout. Indeed, another systematic review (9) revealed that the current state of mental health in HCWs is exacerbated by the role of stress, anxiety, and depression, which can lead to burnout.

Particularly, negative consequences from HCW burnout have been widely reported in healthcare settings during COVID-19, which can include high rates of absenteeism and thoughts of leaving one's current job position. A range of possible causes for absenteeism were identified, including high levels of burnout, poor leadership, job dissatisfaction, lack of childcare, health issues, and job stress (10–13). Specific to burnout, one study (13) reported that 45.8% of physicians reported having a symptom of burnout, and it was directly associated with job satisfaction. Another study (14) that sampled 5,000 actively licensed nurses showed that nurses who had intention to leave their current job were more likely to report burnout and psychological stress. Indeed, increased rates of any type of HCW absenteeism (planned and unplanned) have been reported to negatively affect quality of healthcare services (14, 15). Another recent review on work absenteeism also emphasized the need for support for frontline HCWs, to mitigate elevated levels of burnout as well as staff shortage, particularly during a time of crisis (16). Finally, a scoping review showed that sustainable employment and absenteeism were directly impacted by exacerbated mental health problems as a result of the COVID-19 pandemic (17).

HCWs serve as the foundation of the healthcare system but worker absenteeism and turnover can have significant detrimental impacts on the quality of care and costs to the healthcare system (18, 19). Thus, understanding how burnout and other factors may be independently associated with work absenteeism and intentions to leave work will be important for developing efficacious interventions to reduce deleterious impacts of absenteeism and turnover to the healthcare environment. Existing studies that address work absenteeism during the pandemic are sparse, although a number have reported absenteeism due to COVID-19 in HCWs in low-income countries. This means there is a gap in the literature to fully understand the association between HCW burnout with absenteeism and thoughts of leaving work, specifically during a pandemic (20–23).

In this study, we assessed the cross-sectional association of burnout with work absenteeism and frequency of thoughts of leaving current position in a cohort of HCWs in Illinois during the COVID-19 pandemic.

## Methods

### Study design and participants

**Figure 1:**
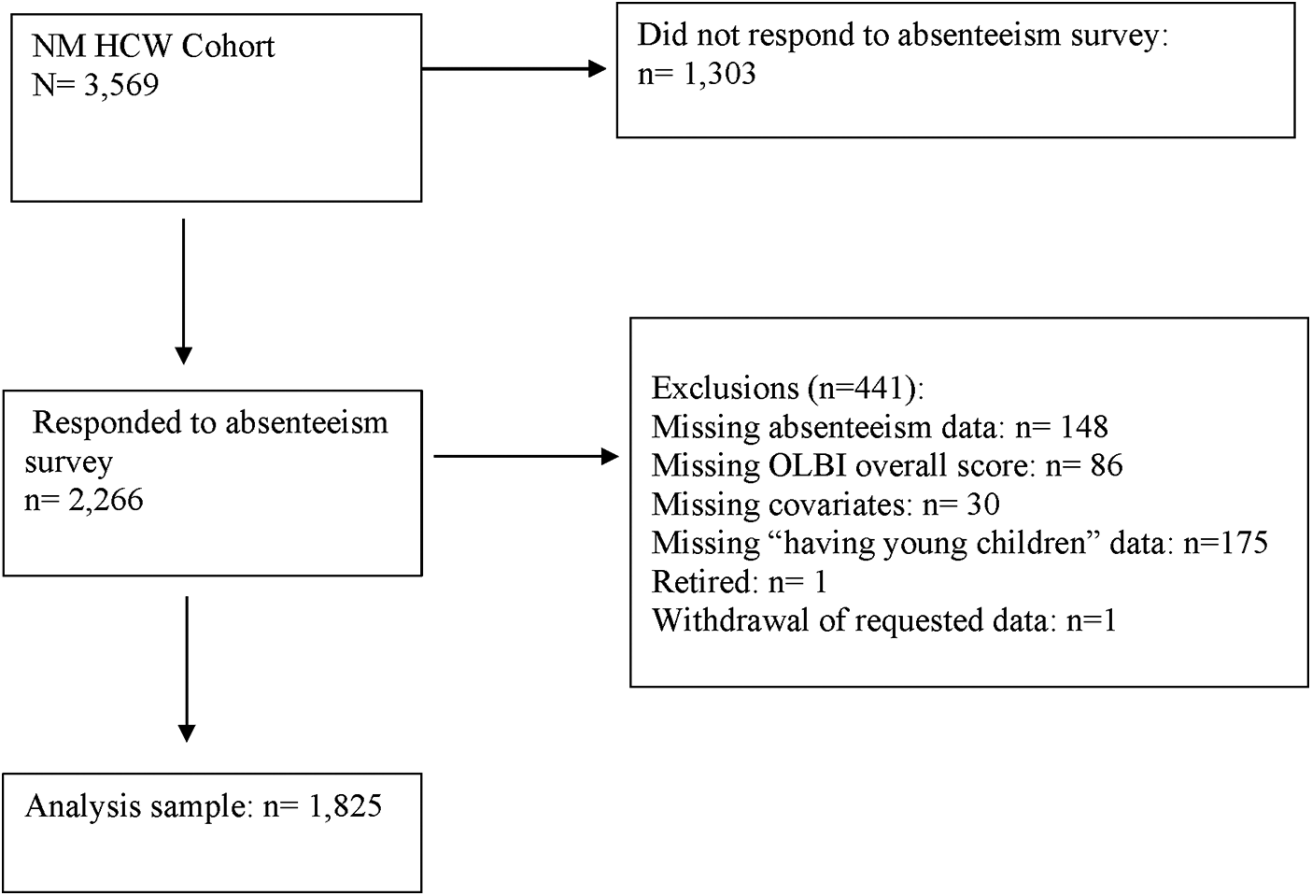
CONSORT diagram for cross-sectional survey between April and May 2022.

A cross-sectional survey was conducted April–May 2022 in a cohort of HCWs currently being followed in a prospective cohort study [Northwestern Medicine (NM) SARS-CoV-2 cohort]. This is a large tertiary academic health care system located in the Chicago area and surrounding suburbs in Illinois. Details of the study and cohort have been previously published (24, 25). Briefly, the cohort includes HCWs from 10 hospitals, 18 immediate care centers, and 325 outpatient practices. Recruitment of the original cohort occurred in May 2020 with 6,510 HCWs enrolled in the study. In June 2021, 3,569 participants re-consented to enroll for continued follow-up (26). In April–May 2022, a survey via REDCap was sent to all 3,569 participants and 2,266 participants completed the survey with an overall response rate of 64.5%. Of 2,266 participants who completed the survey, 441 were excluded due to missing values of variables of interest (*n* = 439), retired (*n* = 1), and withdrawal from the study (*n* = 1) (Figure 1).

### Measures

#### Burnout

Burnout was the main independent variable in this study and measured using the Oldenburg Burnout Inventory (OLBI) (5) a validated 16–item inventory that measures two of these core dimensions of burnout—exhaustion and disengagement (from work). It has strong validity and reliability among English-speaking workers in the United States (5). Sample items include “I always find new and interesting aspects in my work”; “During my work, I often feel emotionally drained”; and “After my work, I usually feel worn out and weary.” Responses to each OLBI item range from 1 (totally disagree) to 4 (totally agree) (5). Thus, the overall Burnout scores range from 16 to 64, and the exhaustion and disengagement (from work) subscale scores range from 8 to 32.

#### Work absenteeism

Absenteeism was the main outcome defined by four questions: “Within the past 6 months, have you been absent from your job?”, “How often have you been absent in the last 6 months?”, “What were the reasons you were absent from work in the last 6 months?”, and “If the main reason was due to own illness, was it due to COVID-19?”. Aligned with previous studies on absenteeism, reasons for absenteeism were classified into planned or unplanned (23, 27). Planned absenteeism was defined as voluntary planned leave, such as annual leave, vacation, study leave, or maternity/paternity leave; whereas unplanned absenteeism was due to involuntary circumstances, such as emergency leave, short-term self-certified sickness absence, medically certified sickness, taking care of a family member, and other family responsibilities (Supplementary Table S1). We used this typology to categorize participants into 2 groups: (1) “Yes” for reporting unplanned absenteeism; and (2) “No” for no unplanned absenteeism. Participants who were in the unplanned only group may have also indicated planned absenteeism in addition to their unplanned absenteeism.

#### Frequency of thoughts of leaving current Job

Similar to absenteeism, the construct of “intention to leave work” has been defined by the CDC (14) and studies of absenteeism (15, 28) as an individual's intention to leave the organization where one is currently employed, within a specific period of time. For this study, this construct was defined as “frequency of thoughts of leaving current job”. Participants were asked: “Within the past 6 months how often have you considered leaving your job?”, in which respondents were grouped by “never/a little” or “a lot/constantly”.

#### Covariates

Participants also self-reported demographics (e.g., age, sex, race/ethnicity), household size, whether they lived with children under the age of 12, and type of occupation at NM. Occupation type was divided into four categories: registered nurse, physician, administrative roles, or other HCWs. Questions on past patient contact (such as taking care of COVID-19 positive patients in person) were also asked.

### Statistical analyses

Respondents to the current survey (*n* = 2,266) were compared to nonrespondents (*n* = 1,303) to determine whether there were any significant differences in participants socio-demographic characteristics. Chi-square tests were conducted for comparisons of proportions for categorical characteristics and *t*-tests for comparison of mean values of continuous characteristics.

For the main analysis, we used binomial logistic regression models to estimate the odds of reporting unplanned absenteeism by burnout score and its sub-categories. Analyses were adjusted for age, sex, race/ethnicity, occupation, patient contact, and “having young children in the household.” A similar adjustment approach was used in multinomial logistic regression models when examining the association between OLBI scores with the frequency of thoughts in leaving current job (with the “never” as the referent). Furthermore, an additional model was conducted to include further adjustment for “unplanned absenteeism.” Two sensitivity analyses were also conducted. Due to COVID-19 illness being associated with mental health (29), we evaluated whether our results changed if HCWs who were absent due to COVID-19 illness were excluded from the analysis.

Furthermore, we encountered missing data in covariates, i.e., sex, occupation, patient contact, and living with young children (*n* = 205) in the study (Figure 1). To ensure the validity and completeness of our analyses, we performed additional sensitivity analysis, using chained equations to obtain 20 multiple imputed datasets to account for all missing values of covariates.

Statistical significance was defined at the *p* < 0.05 level. All analyses were performed using SAS version 9.4 (SAS Institute, Cary, NC, USA).

## Results

The final included sample size for analysis was 1,825 participants. Compared to non-respondents, survey respondents were significantly older (mean age 44.6 vs. 41.5, *p* < 0.001), and more likely to identify as White (81.5% vs. 75.1%, *p* < 0.001). Respondents also were significantly less likely to work as a physician (18.3% vs. 21.1%, *p* < 0.001) or registered nurse (28.6% vs. 33.9%, *p* < 0.001) compared to non-respondents. There was no significant difference in gender of respondents vs. non-respondents (82.2% female vs. 80.2% female) (Table 1).

**Table 1:** Characteristics of the study sample by work absenteeism categories within the past 6 months between May and April 2022.

Characteristics^a^	Overall *N* (%)	Unplanned absenteeism^b^		
		Yes *N* (%)	No *N* (%)	*p*-Value^c^
No. (%)	1,825 (100)	365 (20.0)	1,460 (80.0)	
Age (mean, SD)	44.70 (11.4)	44.82 (10.8)	44.67 (11.6)	0.8
Female	1,508 (82.6)	329 (90.1)	1,179 (80.8)	<0.001
Race/Ethnicity				0.6
Hispanic/Latino	94 (5.2)	22 (6.0)	72 (4.9)	
Asian	163 (8.9)	34 (9.3)	129 (8.8)	
Non-Hispanic White	1,504 (82.4)	293 (803)	1,211 (83)	
Other/NA	64 (3.5)	16 (4.4)	48 (3.3)	
Occupation				0.001
Administrative role	260 (14.3)	36 (9.9)	224 (15.3)	
Registered nurse	520 (28.5)	139 (38.1)	381 (26.1)	
Physician	343 (18.8)	26 (7.1)	317 (21.7)	
Others	702 (38.5)	164 (44.9)	538 (36.9)	
Patient contact within the last month	1,436 (78.7)	311 (85.2)	1,125 (77.1)	0.0007
Living with young children	573 (31.4)	135 (37.0)	438 (30.0)	0.01
Absence due to COVID-19	99 (5.4)	99 (27.1)	0 (0.0)	—

^a^ Numbers are *n* (%) unless otherwise indicated.

^b^ Unplanned absenteeism: any involuntary absence from work, but could also potentially include a planned absence.

^c^ *p*-Values for groups’ comparison based on chi-square test (categorical variables) or *t*-test (continuous variables).

Approximately 20% of the cohort reported unplanned absenteeism (which could also include respondents with planned absences) (Supplementary Table S1). Within unplanned absenteeism, 78.6% were due to illness/injury/medical problems, and 23.8% due to family/personal obligations (Supplementary Table S2). Compared to those with no reports of unplanned absenteeism, unplanned absenteeism participants reported a significant higher proportion of being female, working as a nurse, working without patient contact, or living with younger children (*p* < 0.05) (Table 1). Exhaustion sub-scores were significantly higher in those with reporting unplanned absenteeism compared to those with no unplanned absenteeism in the unadjusted model (19.1 vs. 18.5, *p* = 0.007) (Table 2). Neither unadjusted overall burnout score (36.1 vs. 35.7, *p* = 0.056) or the disengagement subscale score (17.4 vs. 17.2, *p* = 0.42) differed between absenteeism groups (Table 2).

**Table 2:** Burnout and Sub-burnout scores for unplanned absenteeism of the study cohort.

Variable	Overall *N* = 1,825	Unplanned absenteeism^a^		
		Yes *N* = 365	No *N* = 1,460	*p*-Value^b^
OLBI-Overall Burnout score, mean (SD)	35.8 (7.6)	36.5 (7.7)	35.7 (7.57)	0.06
OLBI-Exhaustion score, mean (SD)	18.6 (4.1)	19.1 (4.1)	18.5 (4.1)	0.007
OLBI-Disengagement score, mean (SD)	17.2 (4)	17.4 (4.1)	17.2 (3.9)	0.4

^a^ Unplanned absenteeism: any involuntary absence from work but could also potentially include a planned absence.

^b^ *p*-Values for groups’ comparison based on *F*-tests.

After adjusting for age, race/ethnicity, gender, occupation, patient contact, and having young children in the household, we observed an increase in the odds of reporting unplanned absenteeism for each score increment in overall burnout scores, but the association was only marginally statistically significant: (OR = 1.02, 95% CI: 1.00–1.03, *p* = 0.05). However, being older (OR = 1.02, 95% CI: 1.01–1.03), non-Hispanic Asian (vs. non-Hispanic White) (OR = 1.58, 95% CI: 1.03–2.43), a nurse (OR = 1.65, 95% CI: 1.07–2.54) or “other” job category (vs. administrative job) (OR = 1.58, 90% CI: 1.05–2.40), or having young children in the household (OR = 1.76, 95% CI: 1.34–2.30) were each associated with higher odds of reporting unplanned absenteeism. Male gender (OR = 0.62, 95% CI: 0.42–0.92), being a doctor (vs. administrative job) (OR = 0.35, 95% CI: 0.20–0.62), or work without patient contact (OR = 0.53, 95% CI: 0.38–0.75) had a lower odds of reporting unplanned absenteeism compared to those with no absence (Table 3). The association of exhaustion sub-scores by absenteeism remained after adjustment, (OR = 1.04, 95% CI: 1.01–1.08) (results not tabulated).

**Table 3:** Binomial logistic regression analysis of the association of unplanned absenteeism with burnout (OLBI scores) and covariates.

Effect	Adjusted OR (95%CI)		
	Any unplanned absence^a^ vs. no unplanned absence		
	OR	95% CI	
OLBI-Overall Burnout score	1.02	1.00	1.03
Age	1.02	1.01	1.03
Hispanic vs. non-hispanic white	1.16	0.70	1.93
Non-hispanic Asian vs. non-hispanic white	1.58	1.03	2.43
Non-hispanic others vs. non-hispanic white	1.54	0.84	2.82
Gender: male vs. female	0.62	0.42	0.92
Nurses vs. administrative roles	1.65	1.07	2.54
Doctors vs. administrative roles	0.35	0.20	0.62
Others vs. administrative roles	1.58	1.05	2.40
Patient contact: No vs. Yes	0.53	0.38	0.75
Young children in household: Yes vs. No	1.76	1.34	2.30

^a^ Unplanned absence: any involuntary absence from work but could also potentially include a planned absence.

With frequency of thoughts of leaving one's job, 39.2% reported “never” thinking about leaving one's job, 39.6% reported “a little”, and 21.2% reported “a lot/constantly” thinking of leaving one's job. The frequency of thoughts of leaving one's job varied significantly by age and occupation: those who reported thinking “a lot/constantly” of leaving work were younger than other groups (mean age 42.8 compared to 46.15 and 44.29, *p* < 0.001). For occupation, more than a third (35%) of those who reported thinking “a lot/constantly” about leaving were registered nurses. Additionally, there was no significant difference in the proportion of individuals reporting any unplanned absenteeism based on their levels of thoughts about leaving work (Table 4). The unadjusted burnout score was significantly higher among those who reported frequent thoughts of leaving one's job compared to those with less frequent thoughts of leaving (“a lot/constantly”: 42.7 vs. “a little”: 36.7 vs. “never”: 31.3, *p* < 0.001). This association was the same for both exhaustion (*p* < 0.001) and disengagement subscale scores (*p* < 0.001) (results not tabulated). After adjustment, OLBI-overall burnout scores remained significantly and positively associated with the frequency of thoughts of leaving work (either “a lot/constantly” or “a little” vs. “never”). For example, the odds of reporting thinking about leaving work “a lot/constantly” vs. “never” was 1.39 (95% CI: 1.34–1.43) for each additional burnout score (Table 5). Results were consistent when sub-categories of burnout scores were analyzed. Both exhaustion and disengagement scores also showed a significant positive association with the frequency of thoughts of leaving work as “a lot/constantly” vs. “never”: OR (95% CI) were 1.56 (1.49–1.64) and 1.82 (1.72–1.93), respectively (data not tabled). In addition, being a nurse (vs. administrative job) and having no patient contact were associated with higher odds of reporting thinking about leaving work “a lot/constantly” (Table 5).

**Table 4:** Characteristics of the study sample by frequency of thoughts of leaving work within the past 6 months between May and April 2022.

Characteristics^a^	Overall	Frequency of thoughts in leaving current job category^b^			*p*-Value^c^
		Never	A little	A lot/constantly	
No. (%)	1,825 (100)	716 (39.23)	723 (39.62)	386 (21.15)	
Age (mean, SD)	44.7 (11.4)	46.15 (11.43)	44.29 (11.38)	42.80 (11.13)	<.001
Female	1,508 (82.6)	581 (81.15)	595 (82.30)	332 (86.01)	0.121
Race/Ethnicity					0.650
Hispanic/Latino	94 (5.2)	39 (5.45)	34 (4.70)	21 (5.44)	
Asian	163 (8.9)	74 (10.34)	58 (8.02)	31 (8.03)	
Non-Hispanic White	1,504 (82.4)	581 (81.15)	605 (83.68)	318 (82.38)	
Other/NA	64 (3.5)	22 (3.07)	26 (3.60)	16 (4.15)	
Occupation					0.015
Administrative Role	260 (14.3)	111 (15.50)	107 (14.80)	42 (10.88)	
Registered Nurse	520 (28.5)	174 (24.30)	212 (29.32)	134 (34.72)	
Physician	343 (18.8)	144 (20.11)	130 (17.98)	69 (17.88)	
Others	702 (38.5)	287 (40.08)	274 (37.90)	141 (36.53)	
Patient contact within the last month	1,436 (78.7)	547 (76.40)	579 (80.08)	310 (80.31)	0.158
Living with young children	573 (31.4)	217 (30.31)	242 (33.47)	114 (29.53)	0.292
Unplanned Absence	365 (20.0)	151 (21.1)	140 (19.4)	74 (19.2)	0.644
Total OLBI Burnout Score	35.8 (7.6)	31.3 (6.43)	36.7 (5.84)	42.7 (6.55)	<0.001
Exhaustion Sub-score	18.6 (4.1)	16.5 (3.7)	19.0 (3.41)	21.8 (3.76)	<0.001
Disengagement Sub-score	17.2 (4)	14.9 (3.3)	17.7 (3.56)	20.8 (3.56)	<0.001

^a^ Numbers are *n* (%) unless otherwise indicated.

^b^ Planned absenteeism: voluntary planned absence from work; Unplanned absenteeism: any involuntary absence from work but could also potentially include a planned absence.

^c^ *p*-Values for groups’ comparison based on chi-squared or *F*-tests.

**Table 5:** Multinomial logistic regression analysis of the association between burnout (OLBI scores) and frequency of thoughts of leaving current job.

Effect	A lot/constantly vs. never			A little vs. never		
	OR	95% CI		OR	95% CI	
OLBI-overall Burnout score	1.39	1.34	1.43	1.15	1.13	1.18
Age	1.02	1.00	1.03	1.01	1.00	1.02
Hispanic vs. non-hispanic white	1.53	0.76	3.08	1.03	0.61	1.73
Non-hispanic Asian vs. non-hispanic white	0.68	0.39	1.17	0.70	0.47	1.04
Non-hispanic others vs. non-hispanic white	1.70	0.76	3.81	1.20	0.65	2.24
Gender: male vs. female	1.02	0.65	1.60	1.21	0.88	1.66
Nurses vs. administrative roles	1.99	1.13	3.51	1.05	0.71	1.57
Doctors vs. administrative roles	1.38	0.73	2.61	0.76	0.48	1.18
Others vs. administrative roles	1.36	0.80	2.31	0.87	0.61	1.25
Patient contact: No vs. Yes	1.56	1.02	2.39	1.00	0.73	1.36
Young children in household: Yes vs. No	1.01	0.70	1.42	1.20	0.92	1.55

While there was an association between unplanned absenteeism and intention to leave work, the additional analyses to include further adjustment for “unplanned absenteeism” showed that this association was independent of the relationship between burnout score and intention to leave. For example, the odds of reporting frequent thoughts about leaving work “a lot/constantly” vs. “never” increased by 1.39 (95% CI: 1.35–1.44) for each score increment in burnout. These results were similar to the main analysis results, in which the association was also 1.39 (95% CI: 1.34–1.43). Thus, the results from our analysis adding unplanned absenteeism into the model demonstrated that the relationship between burnout scores and intention to leave work was not attenuated by unplanned absenteeism and remained statistically significant (results not tabulated).

In the first sensitivity analysis (with an exclusion of those who reported their absence was due to a COVID illness), we observed that the effect sizes of burnout sub-scores on unplanned absenteeism were similar to those in the models without the exclusion of COVID-19-related absences in the main analysis. However, the association between the overall burnout scores and unplanned absenteeism became statistically significant (*p* < 0.05) (result not tabulated). In the second sensitivity analysis accounting for missing values of covariates, similar associations/patterns were observed when comparing results from the main analysis with a complete case for both outcomes.

## Discussion

This cohort study surveyed 1,825 HCWs during the COVID-19 pandemic where one-fifth reported unplanned absenteeism and 60% reported thoughts about leaving their jobs. Overall, we observed a marginally significant association in the main analysis, but a significant association was found in both sensitivity analyses. Overall burnout scores were not associated with having unplanned absenteeism. However, burnout subscale of exhaustion scores were found to be significantly associated with reporting unplanned absenteeism, independent from COVID-19 illness status. Further, age, ethnicity, occupation, no patient contact, and having young children in the household—also correlated with higher likelihood of unplanned absenteeism. Furthermore, burnout scores and both sub-categories (exhaustion and disengagement) were significantly associated with higher frequency of thoughts of leaving one's current job.

These findings concur with similar studies that report that HCWs are reporting high levels of burnout at this specific point in time during the pandemic and that burnout could be one factor in explaining high rates of work absenteeism (30–33). The few studies available, however, focus on sick absenteeism (absence due to health reasons), which is consistent with this study's findings. The most recent cross-sectional study by Aiken et al. (34) reported that high stress work conditions were highly associated with burnout and job dissatisfaction, even in workplaces that were deemed “good to work”. Sexton et al. (35) reported significant increases in emotional exhaustion from 31% to 40% in a cohort of HCWs between 2019 and 2021, with only nurses having a steady increase over the 2 years. A cross-sectional survey by Dyrbye et al. (21) reported that 35% of the nurses reported scores that indicated burnout and nurses were more likely to be absent for more than 1 day within the past month, compared to those who were not burned out. It is important to note that the majority of studies available on HCW burnout is focused on nurses. Even prior to the epidemic, a study on nurses in Poland showed that excessive working hours were associated with burnout and higher odds of a sickness absence (36). Outside of sick leave, there were no other studies specific to family obligations, maternity/paternity leave, or conditions where individuals could not find proper work (reasons that were captured in this study).

Specific to work absenteeism, this study found that being a nurse was associated with higher odds of reporting unplanned absenteeism and more frequent thoughts of leaving the work position. Although this may have been because most nurses in this cohort were female, these findings are consistent with the recent U.S. Surgeon General Report, which revealed that nurses, particularly nurses living with young children, had a higher likelihood of reporting unplanned absenteeism. During the pandemic, other similar studies reported that female workers (who were nurses as well as other HCW positions) also reported higher scores of burnout and were more likely than men to face work disruptions as a result of childcare responsibilities (3, 11). Furthermore, the most frequently reported reason for unplanned absenteeism in this cohort was own illness, injury, or others/medical problems (*n* = 287, 15.7%), in which 99 participants reported that it was due to COVID-19. Alongside this, another large proportion of overall participants reported that their absenteeism was a result of family obligations (*n* = 87).

Similarly, a study by Gohar et al. reported that increases of sickness absences in HCWs could be attributed to sickness caused by COVID-19, the flu, injury and/or other health conditions (37). Likewise, another study showed that on average, HCWs took 12.5 absent days after having a COVID-19 infection (38). A study in Turkey (39) showed that there was a total lost time from work of 14,635 days in their HCWs across the span of 1 year (2020–2021). A study in Brazil (40) showed that absenteeism due to a medical reason increased from 633 individuals to 837 from 2019 to 2020, in which nursing assistants were reported to have the highest frequency of medical leave. Other existing literature on HCW absenteeism also reported that sick absenteeism in HCWs was due to a result of COVID-19 vaccine-associated side effects (22, 41). In addition, others reported differing severity in percentages of HCW sickness absenteeism, pending the wave and phase of the COVID-19 pandemic (42). Specific to nurses, Barr (43) reported that 21% of their nurse cohort were considering leaving their job—with burnout and work environment being the top two causes of their intention to leave work.

This study findings also showed that OLBI-overall burnout scores were positively associated with frequency of thoughts to leave work with 21% of this sample reporting thoughts of leaving their job a lot/constantly over the previous 6 months Other studies (pre-COVID-19) have also reported the link between burnout and intention to leave work due to stress (13, 14, 28). LeClaire et al. (44) found that HCWs experiencing burnout had a 50% greater odds of intention to leave work in the next 2 years (defined as likelihood of leaving their practice) than HCWs not experiencing burnout. Literature on the pandemic and rates of absenteeism are primarily international (outside of the US).

A recent burnout framework for HCWs, specific to the pandemic, has listed both absenteeism and intention to leave work as potential sequelae of burnout (31). While frameworks and recommendations exist to reduce burnout in a healthcare setting (3, 20), researchers have yet to provide an extension of a framework for HCWs that targets work absenteeism during extraordinarily stressful times such as a pandemic (16).

The present study is one of very few studies that provides insight in HCWs' experiences of burnout and its association with absenteeism and frequency of thoughts of leaving work in a large cohort of HCWs. However, this study has several limitations. The study is cross-sectional, which limits the ability to determine the causal mechanism linking work absenteeism, frequency of thoughts of leaving work, and burnout. Secondly, there was a lack of information on all previous COVID-19 infections/illnesses in our data, so we were unable to investigate whether the association of interest could be influenced by COVID-19 infection/illness status (or recovery times). However, the association remained similar when we excluded those who reported their absence was due to a COVID-1I illness from the analysis. Furthermore, other studies (45) have also reported that post-COVID-19 patients may also have differing rates of absenteeism and intention to leave work, as a result of slow recovery times. Although our survey did inquire about the reason for absenteeism, we were unable to investigate recovery times and whether intention to work was associated with differing recovery times.

The study findings have important implications for experiences within the HCW workforce during the pandemic. Healthcare systems around the world have faced tremendous stress due to the COVID-19 pandemic. Hence, the findings can provide information on the state of burnout and psychological well-being among HCWs—that could be potentially generalizable to other healthcare organizations during the pandemic. Secondly, as burnout can have negative consequences on healthcare delivery effectiveness and significant cost implications due to medical errors, this study provides insight on how burnout can directly impact HCW absenteeism and future turnover. Maintaining psychological and physical well-being are essential to ensuring a healthy HCW workforce. Thus, the findings of this study indicate how HCWs respond to significant stressors, such as the COVID-19 pandemic, and contribute to lessons learned on how to support HCWs in reducing burnout and ensuring resiliency in the healthcare delivery system.

## Conclusions

In this cohort of HCWs, a significant portion of the HCW cohort reported unplanned absenteeism or were considering leaving their jobs. Notably, the sensitivity analyses showed a statistically significant association between burnout scores and unplanned absenteeism, which was underestimated in the main analyses. We also found that higher levels of burnout were associated with higher frequency of thoughts to leave current position in healthcare. Exhaustion scores were also significantly associated with unplanned absenteeism. Continued examinations of trends in unplanned work absenteeism and frequency of thoughts of leaving work can be useful in fully understanding the impact of post-COVID conditions on burnout. Hence, this study calls for the development of organizational and individual-level programs that can help HCWs cope with the stress of working, particularly during unprecedented times such as COVID-19.

## Data Availability

The dissemination of data results to study participants and or patient organizations in this research project is not available nor possible due to restrictions that are applied to the public availability of these data and our data access agreements for the current study. Requests to access the datasets should be directed to hcwstudy@northwestern.edu.

## References

[1] Zareei M Tabanejad Z Oskouie F Ebadi A Mesri M. Job burnout among nurses during COVID-19 pandemic: a systematic review. J Educ Health Promot. (2022) 11:107. 10.4103/jehp.jehp_797_21 35573618 PMC9093652

[2] Wei H Aucoin J Kuntapay GR Justice A Jones A Zhang C The prevalence of nurse burnout and its association with telomere length pre and during the COVID-19 pandemic. PLoS ONE. (2022) 17(3):e0263603. 10.1371/journal.pone.0263603 35294438 PMC8926201

[3] Surgeon General US. Addressing Health Worker Burnout. Public Health Service (2022). Available at: https://www.hhs.gov/sites/default/files/health-worker-wellbeing-advisory.pdf

[4] Rossi MF Gualano MR Magnavita N Moscato U Santoro PE Borrelli I. Coping with burnout and the impact of the COVID-19 pandemic on workers’ mental health: a systematic review. Front Psychiatry. (2023) 14:1139260. 10.3389/fpsyt.2023.1139260 37009102 PMC10060559

[5] Maslach C Leiter MP. Understanding the burnout experience: recent research and its implications for psychiatry. World Psychiatry. (Jun 2016) 15(2):103–11. 10.1002/wps.20311 27265691 PMC4911781

[6] Profit J Sharek PJ Amspoker AB Kowalkowski MA Nisbet CC Thomas EJ Burnout in the NICU setting and its relation to safety culture. BMJ Qual Saf. (Oct 2014) 23(10):806–13. 10.1136/bmjqs-2014-002831 24742780 PMC4167972

[7] Salvagioni DAJ Melanda FN Mesas AE Gonzalez AD Gabani FL Andrade SM. Physical, psychological and occupational consequences of job burnout: a systematic review of prospective studies. PLoS ONE. (2017) 12(10):e0185781. 10.1371/journal.pone.0185781 28977041 PMC5627926

[8] Bannon J Evans CT Freedman M Lee C Vu TH Wallia A Psychological wellbeing and the association with burnout in a cohort of healthcare workers during the COVID-19 pandemic. Front Health Serv. (2022) 2:994474. 10.3389/frhs.2022.994474 36925776 PMC10012723

[9] Salari N Khazaie H Hosseinian-Far A Khaledi-Paveh B Kazeminia M Mohammadi M The prevalence of stress, anxiety and depression within front-line healthcare workers caring for COVID-19 patients: a systematic review and meta-regression. Hum Resour Health. (2020) 18(1):100. 10.1186/s12960-020-00544-1 33334335 PMC7745176

[10] Bordignon M Monteiro MI. Predictors of nursing workers’ intention to leave the work unit, health institution and profession. Rev Lat Am Enfermagem. (2019) 27:e3219. 10.1590/1518-8345.3280.3219 31826161 PMC6896814

[11] Dall'Ora C Ball J Reinius M Griffiths P. Burnout in nursing: a theoretical review. Hum Resour Health. (2020) 18(1):41. 10.1186/s12960-020-00469-9 32503559 PMC7273381

[12] Yue Z Qin Y Li Y Wang J Nicholas S Maitland E Empathy and burnout in medical staff: mediating role of job satisfaction and job commitment. BMC Public Health. (2022) 22(1):1033. 10.1186/s12889-022-13405-4 35606769 PMC9125814

[13] Shanafelt TD Boone S Tan L Dyrbye LN Sotile W Satele D Burnout and satisfaction with work-life balance among US physicians relative to the general US population. Arch Intern Med. (2012) 172(18):1377–85. 10.1001/archinternmed.2012.3199 22911330

[14] Han K Trinkoff AM Gurses AP. Work-related factors, job satisfaction and intent to leave the current job among United States nurses. J Clin Nurs. (2015) 24(21–22):3224–32. 10.1111/jocn.12987 26417730

[15] Kivimaki M Vanhala A Pentti J Länsisalmi H Virtanen M Elovainio M Team climate, intention to leave and turnover among hospital employees: prospective cohort study. BMC Health Serv Res. (2007) 7:170. 10.1186/1472-6963-7-170 17956609 PMC2190768

[16] Tujjar O Simonelli M. Absenteeism of frontline healthcare workers during COVID-19: the need for a framework of support. SN Compr Clin Med. (2020) 2(12):2715–7. 10.1007/s42399-020-00609-1 33134848 PMC7592146

[17] van den Broek A van Hoorn L Tooten Y de Vroege L. The moderating effect of the COVID-19 pandemic on the mental wellbeing of health care workers on sustainable employability: a scoping review. Front Psychiatry. (2022) 13:1067228. 10.3389/fpsyt.2022.1067228 36683992 PMC9852887

[18] Hewitt DB Ellis RJ Chung JW Cheung EO Moskowitz JT Huang R Association of surgical resident wellness with medical errors and patient outcomes. Ann Surg. (2021) 274(2):396–402. 10.1097/SLA.0000000000003909 32282379

[19] Panagioti M Geraghty K Johnson J Zhou A Panagopoulou E Chew-Graham C Association between physician burnout and patient safety, professionalism, and patient satisfaction: a systematic review and meta-analysis. JAMA Intern Med. (2018) 178(10):1317–31. 10.1001/jamainternmed.2018.3713 30193239 PMC6233757

[20] De Hert S. Burnout in healthcare workers: prevalence, impact and preventative strategies. Local Reg Anesth. (2020) 13:171–83. 10.2147/LRA.S240564 33149664 PMC7604257

[21] Dyrbye LN Shanafelt TD Johnson PO Johnson LA Satele D West CP. A cross-sectional study exploring the relationship between burnout, absenteeism, and job performance among American nurses. BMC Nurs. (2019) 18:57. 10.1186/s12912-019-0382-7 31768129 PMC6873742

[22] Maltezou HC Panagopoulos P Sourri F Giannouchos TV Raftopoulos V Gamaletsou MN COVID-19 vaccination significantly reduces morbidity and absenteeism among healthcare personnel: a prospective multicenter study. Vaccine. (2021) 39(48):7021–7. 10.1016/j.vaccine.2021.10.054 34740473 PMC8556541

[23] Belita A Mbindyo P English M. Absenteeism amongst health workers–developing a typology to support empiric work in low-income countries and characterizing reported associations. Hum Resour Health. (2013) 11:34. 10.1186/1478-4491-11-34 23866770 PMC3721994

[24] Wilkins JT Hirschhorn LR Gray EL Wallia A Carnethon M Zembower TR Serologic status and SARS-CoV-2 infection over 6 months of follow up in healthcare workers in Chicago: a cohort study. Infect Control Hosp Epidemiol. (2022) 43(9):1207–15. 10.1017/ice.2021.367 34369331 PMC8438416

[25] Wilkins JT Gray EL Wallia A Hirschhorn LR Zembower TR Ho J Seroprevalence and correlates of SARS-CoV-2 antibodies in health care workers in Chicago. Open Forum Infect Dis. (2021) 8(1):ofaa582. 10.1093/ofid/ofaa582 33447642 PMC7787182

[26] Evans CT DeYoung BJ Gray EL Wallia A Ho J Carnethon M Coronavirus disease 2019 (COVID-19) vaccine intentions and uptake in a tertiary-care healthcare system: a longitudinal study. Infect Control Hosp Epidemiol. (2022) 43(12):1806–12. 10.1017/ice.2021.523 34955103 PMC8770845

[27] Mat Saruan NA Mohd Yusoff H Mohd Fauzi MF Wan Puteh SE Muhamad Robat R. Unplanned absenteeism: the role of workplace and non-workplace stressors. Int J Environ Res Public Health. (2020) 17(17):1–16. 10.3390/ijerph17176132 PMC7504706 32846878

[28] Ayalew E Workineh Y Semachew A Woldgiorgies T Kerie S Gedamu H Nurses’ intention to leave their job in Sub-Saharan Africa: a systematic review and meta-analysis. Heliyon. (2021) 7(6):e07382. 10.1016/j.heliyon.2021.e07382 34258453 PMC8253915

[29] Taquet M Geddes JR Husain M Luciano S Harrison PJ. 6-month neurological and psychiatric outcomes in 236 379 survivors of COVID-19: a retrospective cohort study using electronic health records. Lancet Psychiatry. (2021) 8(5):416–27. 10.1016/S2215-0366(21)00084-5 33836148 PMC8023694

[30] Marvaldi M Mallet J Dubertret C Moro MR Guessoum SB. Anxiety, depression, trauma-related, and sleep disorders among healthcare workers during the COVID-19 pandemic: a systematic review and meta-analysis. Neurosci Biobehav Rev. (2021) 126:252–64. 10.1016/j.neubiorev.2021.03.024 33774085 PMC9754720

[31] Maunder RG Heeney ND Hunter JJ Strudwick G Jeffs LP Ginty L Trends in burnout and psychological distress in hospital staff over 12 months of the COVID-19 pandemic: a prospective longitudinal survey. J Occup Med Toxicol. (2022) 17(1):11. 10.1186/s12995-022-00352-4 35614505 PMC9132565

[32] Shechter A Norful AA. A peripandemic examination of health care worker burnout and implications for clinical practice, education, and research. JAMA Netw Open. (2022) 5(9):e2232757. 10.1001/jamanetworkopen.2022.32757 36129714 PMC10688030

[33] Parent-Lamarche A Laforce S. A moderated mediation analysis of new work-related stressors, psychological distress, and absenteeism in health care during a pandemic: is recognition the cure for preventing falling in battle? J Occup Environ Med. (2022) 64(10):839–47. 10.1097/JOM.0000000000002619 35901202 PMC9524531

[34] Aiken LH Lasater KB Sloane DM Pogue CA Fitzpatrick Rosenbaum KE Muir KJ Physician and nurse well-being and preferred interventions to address burnout in hospital practice: factors associated with turnover, outcomes, and patient safety. JAMA Health Forum. (2023) 4(7):e231809. 10.1001/jamahealthforum.2023.1809 37418269 PMC10329209

[35] Sexton JB Adair KC Proulx J Profit J Cui X Bae J Emotional exhaustion among US health care workers before and during the COVID-19 pandemic, 2019–2021. JAMA Netw Open. (2022) 5(9):e2232748. 10.1001/jamanetworkopen.2022.32748 36129705 PMC9494188

[36] Kowalczuk K Krajewska-Kulak E Sobolewski M. Working excessively and burnout among nurses in the context of sick leaves. Front Psychol. (2020) 11:285. 10.3389/fpsyg.2020.00285 32158416 PMC7052176

[37] Gohar B Lariviere M Nowrouzi-Kia B. Sickness absence in healthcare workers during the COVID-19 pandemic. Occup Med (Lond). (2020) 70(5):338–42. 10.1093/occmed/kqaa093 32449751 PMC7313824

[38] Sakr CJ Fakih L Melhem NM Fakhreddine M Musharrafieh U Banna H COVID-19 infections and predictors of sickness related absences among healthcare workers: the experience of a tertiary care center with the COVID-19 pandemic. J Occup Environ Med. (2023) 65(7):590–4. 10.1097/JOM.0000000000002857 37015731 PMC10332510

[39] Pirdal BZ Toplu FS Esen BK Aydin SN Erginoz E Can G. An assessment on loss of workforce due to COVID-19 among healthcare personnel: a university hospital experience. Work. (2022) 73(1):59–67. 10.3233/WOR-211308 35912781

[40] Garbin AJI Nascimento C Zacharias FCM Garbin CAS Moimaz SAS Saliba NA. Sickness absenteeism of primary health care professionals before and during the COVID-19 pandemic. Rev Bras Enferm. (2022) 75(Suppl 1):e20220028. 10.1590/0034-7167-2022-0028 36043603

[41] Chrissian AA Oyoyo UE Patel P Lawrence Beeson W Loo LK Tavakoli S Impact of COVID-19 vaccine-associated side effects on health care worker absenteeism and future booster vaccination. Vaccine. (2022) 40(23):3174–81. 10.1016/j.vaccine.2022.04.046 35465979 PMC9013647

[42] Al-Nuaimi AA Abdeen S Abed Alah M AlHajri S Semaan S Al-Kuwari MG. Sickness absenteeism among primary health care workers in Qatar before and during the COVID-19 pandemic. J Occup Med Toxicol. (2023) 18(1):3. 10.1186/s12995-023-00369-3 36927778 PMC10018637

[43] Barr P. Moral distress and considering leaving in NICU nurses: direct effects and indirect effects mediated by burnout and the hospital ethical climate. Neonatology. (2020) 117(5):646–9. 10.1159/000509311 32750693

[44] LeClaire M Poplau S Linzer M Brown R Sinsky C. Compromised integrity, burnout, and intent to leave the job in critical care nurses and physicians. Crit Care Explor. (2022) 4(2):e0629. 10.1097/CCE.0000000000000629 35156049 PMC8824411

[45] Buonsenso D Gualano MR Rossi MF Valz Gris A Sisti LG Borrelli I Post-acute COVID-19 sequelae in a working population at one year follow-up: a wide range of impacts from an Italian sample. Int J Environ Res Public Health. (2022) 19(17):1–12. 10.3390/ijerph191711093 PMC9518581 36078808

