# Supplementary material for: The association of burnout with work absenteeism and the frequency of thoughts in leaving their job in a cohort of healthcare workers during the COVID-19 pandemic
Source: Front Health Serv. 2023 Nov 29;3:1272285. doi: 10.3389/frhs.2023.1272285 (PMC10716445; doi:10.3389/frhs.2023.1272285)
Supplement: Supplementary file 1 [file Table1.docx]

**Supplementary Materials**

**Supplemental Table 1: Reasons for work absenteeism categorized into planned and unplanned (n=1,825)**

| **Categorizations and Reason for Work Absenteeism** | **(n, %)** |
| --- | --- |
| ***Planned Absenteeism*** | |
| Vacation/personal days | 229 (12.6) |
| Maternity/paternity leave | 26 (1.4) |
| School/training | 5 (0.3) |
| Waiting for new job to begin | 5 (0.3) |
| Civic/military duty | 2 (0.1) |
| ***Unplanned absenteeism*** | |
| Own illness/injury/medical problems | 287 (15.7) |
| Childcare | 68 (3.7) |
| Family/personal obligation | 87 (4.8) |
| Weather affected job | 7 (0.4) |
| Slack work/business conditions | 4 (0.2) |
| On layoff (temporary or indefinite) | 1 (0.1) |
| Labor dispute | 0 |

**Supplemental Table 2: Reasons for work absenteeism among unplanned absences(n=365)**

| **Reasons** |  |
| --- | --- |
| Own illness/injury/medical problems | 287 (78.6) |
| Childcare | 68 (18.6) |
| Family/personal obligation | 87 (23.8) |
| Weather affected job | 7 (1.9) |
| Slack work/business conditions | 4 (1.1) |
| On layoff (temporary or indefinite) | 1 (0.3) |
| Labor dispute | 0 |

**Supplemental Table 3. Binomial logistic regression analysis of the association of unplanned absenteeism with burnout (OLBI scores) and covariates- results from 20 imputations of the covariates using chained equations**

| **Effect** | **OR** | **95% CI** | | **P_value** |
| --- | --- | --- | --- | --- |
| OLBI-Overall Burnout score | 1.02 | 1.01 | 1.03 | 0.042 |
| Age | 1.02 | 1.01 | 1.03 | 0.001 |
| Hispanic versus non-hispanic white | 1.30 | 0.81 | 2.09 | 0.271 |
| Non-hispanic Asian versus non-hispanic white | 1.59 | 1.05 | 2.39 | 0.027 |
| Non-hispanic others versus non-hispanic white | 1.28 | 0.73 | 2.24 | 0.383 |
| Gender: male versus female | 0.61 | 0.42 | 0.88 | 0.009 |
| Nurses versus administrative roles | 1.60 | 1.06 | 2.43 | 0.027 |
| Doctors versus administrative roles | 0.37 | 0.22 | 0.65 | <0.001 |
| Others versus administrative roles | 1.65 | 1.11 | 2.44 | 0.013 |
| Patient contact: no versus yes | 0.58 | 0.42 | 0.80 | <0.001 |
| Young children in household: yes versus no | 1.73 | 1.32 | 2.26 | <0.001 |

**Supplemental Table 4. Multinomial logistic regression analysis of the association between burnout (OLBI overall scores) and frequency of thoughts of leaving current job- results from 20 imputations of the covariates using chained equations**

| **Effect** | **A lot/constantly vs. never** | | | **A little vs. never** | | |
| --- | --- | --- | --- | --- | --- | --- |
|  | **OR** | **95% CI** | | **OR** | **95% CI** | |
| OLBI-Overall Burnout score | 1.39 | 1.35 | 1.43 | **1.15** | **1.13** | **1.18** |
| Age | 1.02 | 1.003 | 1.03 | 1.01 | 0.998 | 1.02 |
| Hispanic versus non-hispanic white | 1.61 | 0.82 | 3.17 | 1.23 | 0.76 | 2.00 |
| Non-hispanic Asian versus non-hispanic white | 0.73 | 0.43 | 1.24 | 0.76 | 0.52 | 1.11 |
| Non-hispanic others versus non-hispanic white | 1.43 | 0.68 | 2.98 | 1.19 | 0.69 | 2.07 |
| Gender: male versus female | 1.07 | 0.70 | 1.63 | 1.23 | 0.91 | 1.65 |
| Nurses versus administrative roles | 1.90 | 1.11 | 3.28 | 1.05 | 0.72 | 1.54 |
| Doctors versus administrative roles | 1.32 | 0.72 | 2.43 | 0.70 | 0.46 | 1.06 |
| Others versus administrative roles | 1.31 | 0.79 | 2.18 | 0.85 | 0.60 | 1.19 |
| Patient contact: no versus yes | 1.55 | 1.03 | 2.32 | 0.98 | 0.73 | 1.31 |
| Young children in household: yes versus no | 1.00 | 0.70 | 1.42 | 1.17 | 0.91 | 1.52 |
